# Alternating Differentiation and Dedifferentiation between Mature Osteoblasts and Osteocytes

**DOI:** 10.1038/s41598-019-50236-7

**Published:** 2019-09-25

**Authors:** Naruhiko Sawa, Hiroki Fujimoto, Yoshihiko Sawa, Junro Yamashita

**Affiliations:** 1 Department of Oral Rehabilitation, Fukuoka Dental College, Fukuoka, Japan; 2 Department of Oral Function and Anatomy, Okayama University Graduate School of Medicine, Dentistry and Pharmaceutical Sciences, Okayama, Japan; 3 Center for Regenerative Medicine, Fukuoka Dental College, Fukuoka, Japan

**Keywords:** Biochemistry, Cell biology

## Abstract

Osteocytes are terminally differentiated osteoblasts embedded in the bone matrix. Evidence indicates that cells in the mesenchymal lineage possess plasticity. However, whether or not osteocytes have the capacity to dedifferentiate back into osteoblasts is unclear. This study aimed to clarify the dedifferentiation potential of osteocytes. Mouse calvarial osteoblasts were isolated and maintained in normal two-dimensional (2D) or collagen gel three-dimensional (3D) cultures. In 2D cultures, osteoblasts exhibited a typical fibroblast-like shape with high *Alpl* and minimal *Sost*, *Fgf23*, and *Dmp1* expression and osteoblasts formed mineralised nodules. When these osteoblasts were transferred into 3D cultures, they showed a stellate shape with diminished cytoplasm and numerous long processes and expression of *Alpl* decreased while *Sost*, *Fgf23*, and *Dmp1* were significantly increased. These cells were in cell cycle arrest and showed suppressed mineralisation, indicating that they were osteocytes. When these osteocytes were recovered from 3D cultures and cultured two-dimensionally again, they regained adequate cytoplasm and lost the long processes, resulting in a fibroblast-like shape. These cells showed high *Alpl* and low *Sost*, *Fgf23*, and *Dmp1* expression with a high mineralisation capability, indicating that they were osteoblasts. This report shows that osteocytes possess the capacity to dedifferentiate back into mature osteoblasts without gene manipulation.

## Introduction

Osteocytes, which reside within lacunae of mineralised matrix in bone, have long been thought to be terminally differentiated cells derived from mature osteoblasts. The biological mechanisms by which osteoblasts differentiate into osteocytes have been previously reported^1^. However, the fate of osteocytes—whether or not they dedifferentiate back into osteoblasts when liberated from the mineralised matrix by bone resorption—has been unclear.

The literature indicates that mature osteoblasts go through a progressive transformation to osteocytes, driven by substantial changes in gene expression^2–4^. As the differentiation advances, cells undergo striking morphological changes from a cuboidal to stellate shape with multiple neuron-like cytoplasmic dendrites that interconnect with the dendrites from neighbouring osteocytes and cells on the bone surface^5,6^. The cytoplasm diminishes considerably during differentiation^7^. Concurrently, the expression of alkaline phosphatase (*Alpl*), an osteoblast marker gene, decreases^8,9^, while osteocyte marker gene expression, especially sclerostin (*Sost*), dentin matrix protein 1 (*Dmp1*), and fibroblast growth factor 23 (*Fgf23*), increases^4,10^. Furthermore, osteocytes live markedly longer than osteoblasts, with an estimated survival of about three months for osteoblasts versus decades for osteocytes^11^. During this time, osteocytes are in cell cycle arrest and therefore no longer undergo mitosis.

Bone remodelling takes place when damaged or old bone requires repair. Osteoclasts initiate bone resorption in response to signalling through the osteocyte lacuna-canaliculi network. A cutting cone cavity is formed and moves towards the damaged site. During this process, some healthy bone matrix is subjected to osteoclastic resorption, and osteocytes in the affected vicinity are released^12,13^. Although such released osteocytes are typically phagocytosed by osteoclasts, they can also survive and potentially participate in bone repair^14,15^. However, little is known about whether or not osteocytes have the capacity to dedifferentiate back into mature osteoblasts.

The present study explored whether or not osteocytes are able to dedifferentiate back into mature osteoblasts without gene manipulation *in vitro*.

## Results

### Drastic changes in cell morphology occurred, depending on culture conditions

**Figure 1:**
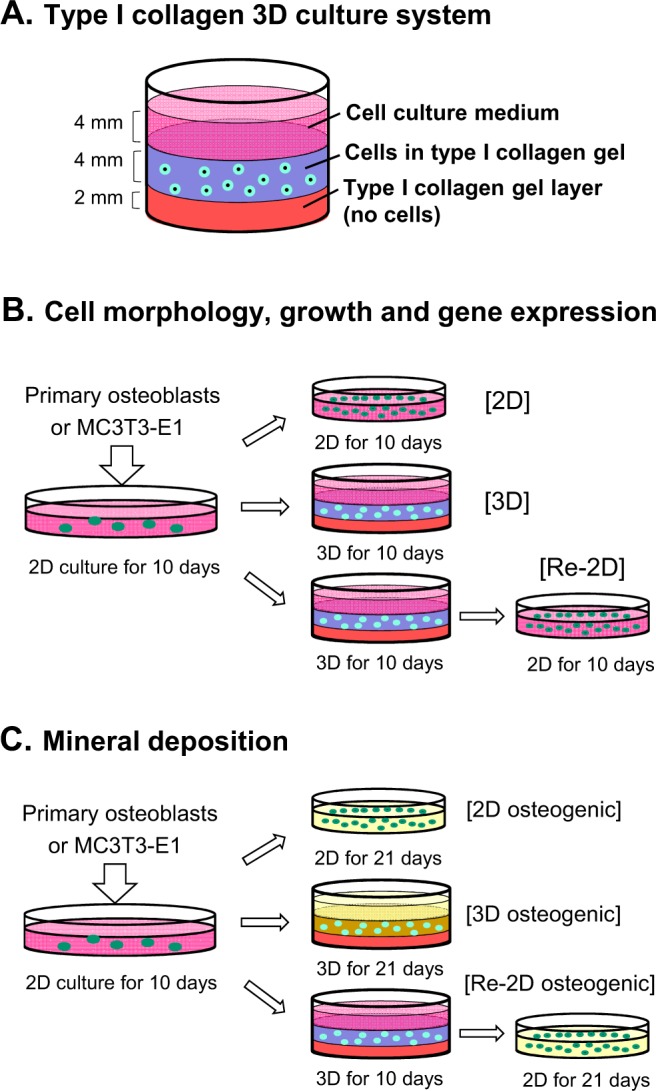
Experimental design. (**A**) The three-dimensional (3D) culture system consists of three layers: a collagen gel layer (bottom), the collagen gel layer containing cells (middle), and the culture medium layer (top). (**B**) Isolated primary osteoblasts or MC3T3-E1 cells were grown in 2-dimensional (2D) cultures with the normal medium for 10 days. The cells were trypsinized, washed, counted, and re-plated in either 2D or 3D cultures for 10 days. A separate group of cells in 3D cultures for 10 days was recovered and plated back in 2D cultures for another 10 days (Re-2D). These cell cultures were used for the assessment of the cell morphology, proliferation, and gene expression. (**C**) Isolated primary osteoblasts or MC3T3-E1 cells were grown in 2D cultures with normal medium for 10 days. The cells were recovered and re-plated in either 2D or 3D cultures in osteogenic medium (yellow) for 3 weeks. A separate group of cells in 3D cultures with normal medium for 10 days was recovered and plated back in 2D cultures with osteogenic medium (yellow) for another 21 days (Re-2D). These osteogenic cultures were used for the assessment of calcium deposition.

**Figure 2:**
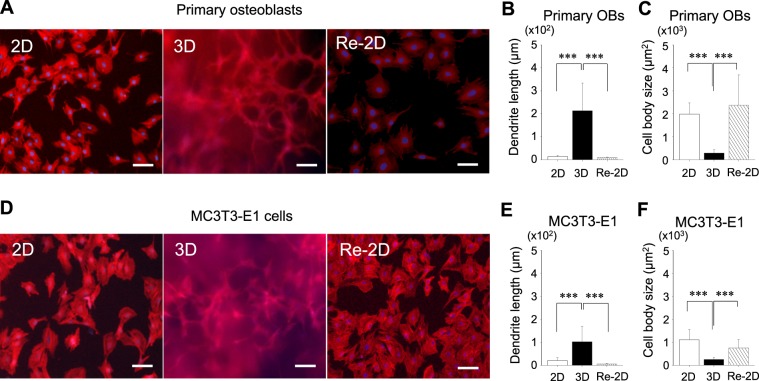
Characterisation of the cell morphology. Representative fluorescent images of primary osteoblasts (**A**) and MC3T3-E1 cells (**D**) stained with DAPI for cell nuclei (blue) and rhodamine-phalloidin for actin filaments (red) (bar: 100 µm). Cells were cultured for 10 days under 2-dimensional (2D) or 3-dimensional (3D) conditions. A separate group of cells in 3D cultures for 10 days was recovered and plated back in 2D cultures for another 10 days (Re-2D). The dendrites and cell bodies of primary osteoblasts (**B**,**C**) and MC3T3-E1 cells (**E**,**F**) were morphometrically assessed and compared between 2D, 3D, and Re-2D cultures. For each culture, at least 20 cells were randomly selected and morphometrically analysed. The graphs show the mean values ± standard deviation of three independent experiments. Data were statistically analysed using a one-way ANOVA. ***p < 0.001.

Primary osteoblasts and MC3T3-E1 cells were cultured in either 2-dimensional (2D) or 3-dimensional (3D) conditions for 10 days (Fig. 1). Cells grown in 3D cultures were recovered and plated in 2D conditions for another 10 days (Re-2D). To investigate cell differentiation/dedifferentiation between osteoblasts and osteocytes, cells were morphologically characterized. Cells were fluorescently visualized using phalloidin and DAPI (Fig. 2A,D). When primary osteoblasts were grown in 2D cultures, the conventional *in vitro* cell culture method in biology, cells exhibited a typical fibroblast-like morphology with relatively short dendritic extensions anchoring the cells to the dish surface. The cells appeared to have adequate plump cytoplasm (Fig. 2A left). However, when cells were grown in 3D cultures, cell morphology drastically changed to a stellate appearance, which is characterized by numerous cytoplasmic long dendrites radiating randomly (Fig. 2A middle). The dendrites appeared to be connected to other dendrites projected from other cells. When these stellate-shaped cells were recovered and subjected to Re-2D plating, approximately 70–80% of cells survived at day 1 and proliferated thereafter. The stellate appearance disappeared, and the cells exhibited a typical fibroblast-like morphology, presenting with adequate cytoplasm with short dendritic extensions (Fig. 2A right).

Morphometric analyses revealed that the dendrites of the cells grown in 3D cultures were significantly longer than those in 2D and Re-2D cultures (Fig. 2B). Furthermore, the body size of the cells in 3D cultures was significantly smaller than that in 2D and Re-2D cultures (Fig. 2C). However, no marked differences were noted in the dendrite length or body size of cells between 2D and Re-2D.

When MC3T3-E1 cells were grown in 2D, 3D, and Re-2D cultures, the cell morphology changed similarly as primary osteoblasts (Fig. 2D). MC3T3-E1 cells showed a fibroblast-like shape in 2D cultures and a stellate appearance with numerous long dendrites in 3D cultures (Fig. 2D left and middle). When such stellate-shaped cells were recovered from 3D cultures and plated back into 2D cultures (Re-2D), MC3T3-E1 cells presented in the fibroblast-like morphology (Fig. 2D right). Indeed, the dendrite length of cells in 3D cultures was significantly longer than that in 2D and Re-2D cultures (Fig. 2E). Furthermore, the cell size was significantly smaller in 3D than that in 2D and Re-2D cultures (Fig. 2F). When the dendrite length of stellate-shaped cells in 3D cultures was compared between primary and MC3T3-E1 cells, significantly longer dendrites were noted in primary cells than those in MC3T3-E1 cells (*p* = 2.2E-05). No marked differences were found in the cell body size between these two cell types. These findings suggest that cell morphology shifts between fibroblast-like and stellate shapes, depending on the culture condition (2D or 3D, respectively).

### The expression of osteocyte-specific genes increased in 3D and decreased in Re-2D cultures

To assess the expression of osteoblast- and osteocyte-specific genes in cells from different culture conditions, qPCR was performed. The expression of *Alpl*, an osteoblast marker, was quantified in order to assess the osteoblastic phenotype of cells, whereas the expression of *Sost*, *Fgf23*, and *Dmp1*, which are osteocyte markers, was quantified to assess the osteocytic phenotype of cells. *Sp7* (*Osterix*), which is expressed in both osteoblasts and osteocytes, was also assessed. In addition, the expression of *Tnfsf11* (*Rankl*) and *Tnfrsf11b* (*Opg*) was analysed. It was confirmed that the *Gapdh* expression was similar between 2D, 3D, and Re-2D conditions (Supplementary Fig. S1).

**Figure 3:**
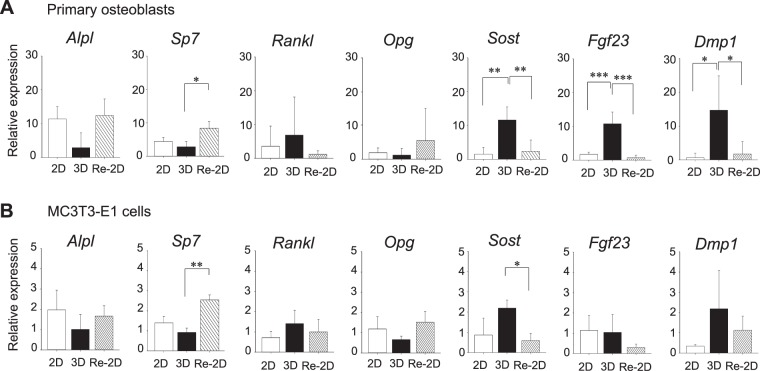
Gene expression profiles of osteoblast- and osteocyte-specific markers. Primary osteoblasts (**A**) and MC3T3-E1 cells (**B**) were cultured for 10 days under 2-dimensional (2D) or 3-dimensional (3D) conditions. A separate group of cells in 3D cultures for 10 days were recovered and plated back in 2D cultures for another 10 days (Re-2D). The expression of *Alpl*, *Sp7*, *Sost*, *Fgf23*, *Dmp1*, *Rankl*, and *Opg* was determined by quantitative PCR. The relative expression of each gene to the internal control was calculated and compared between 2D, 3D, and Re-2D cultures. The graphs show the mean values ± standard deviation of at least three independent experiments. Data were statistically analysed using a one-way ANOVA. *p < 0.05; **p < 0.01; ***p < 0.001.

In primary cells (Fig. 3A), the expression of *Alpl* increased after 10 days in 2D cultures but not in 3D cultures. When the stellate-shaped cells in 3D cultures were recovered and plated back in 2D cultures (Re-2D) for another 10 days, a strong expression of *Alpl* was noted. The expression of osteocyte-specific genes*—Sost, Fgf23*, and *Dmp1—*was low when primary cells were grown in 2D cultures. However, when cells were maintained in 3D cultures for 10 days, a significant increase in the *Sost*, *Fgf23*, and *Dmp1* expression was observed. When the stellate-shaped cells in 3D cultures were recovered and plated back in 2D cultures (Re-2D), cells again expressed very low levels of *Sost, Fgf23*, and *Dmp1*. No significant difference was found in the *Sp7* expression in cells between 2D and 3D cultures; however, when the stellate-shaped cells in 3D cultures were recovered and plated back in 2D cultures (Re-2D), *Sp7* expression significantly increased. These gene expression profiles suggest that cells in 2D cultures align with an osteoblastic phenotype, the stellate-shaped cells in 3D cultures align with an osteocytic phenotype, and that cells in Re-2D culture returned to the osteoblastic state. No recognisable regulation of *Rankl* or *Opg* was observed when cells were cultured in 2D, 3D, and Re-2D conditions.

MC3T3-E1 cells also showed a similar gene expression pattern to that of primary osteoblasts (Fig. 3B), although no marked differences were noted in the *Fgf23* expression in cells between 2D, 3D, and Re-2D cultures. In MC3T3-E1 cells, the levels of respective gene expressions were much lower than those of the primary osteoblasts.

### Cells are proliferative in 2D and Re-2D cultures, but not in 3D cultures

**Figure 4:**
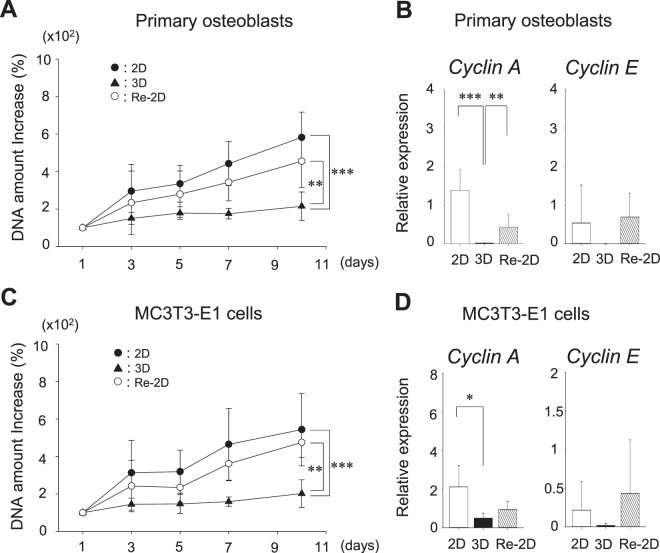
The cell proliferation and gene expression of *Cyclin A* and *E*. Cells were cultured for 10 days under 2-dimensional (2D) or 3-dimensional (3D) conditions. A separate group of cells in 3D cultures for 10 days was recovered and plated back in 2D cultures for another 10 days (Re-2D). The total DNA amount was measured at days 1, 3, 5, 7, and 10 as a surrogate of cell proliferation in primary osteoblast cultures (**A**) and MC3T3-E1 cultures (**C**). The change of the total DNA amount was compared between 2D, 3D, and Re-2D using a two-way ANOVA. Cells at day 10 were harvested. The expression of *Ccna2* (*Cyclin A)* and *Ccne1* (*Cyclin E)* was determined by quantitative PCR (**B**,**D**). The relative expression of each gene to the internal control was calculated and compared between 2D, 3D, and Re-2D cultures. The graphs show the mean values ± standard deviation of at least three independent experiments. Data were statistically analysed using a one-way ANOVA. **p < 0.01; ***p < 0.001.

To determine any differences in cell proliferation, total DNAs were collected from cells at days 1, 3, 5, 7, and 10, and the amounts of DNA were plotted (Fig. 4A,C), with the DNA amount used as a surrogate for the accumulated cell proliferation activity.

The amount of DNA was increased in the 2D cultures of primary osteoblasts, while the amount of DNA in the 3D cultures (primary osteocytes) were not significantly changed over time. However, when cells in 3D cultures were recovered and plated back in 2D cultures (Re-2D) for another 10 days, the increase over time of DNA amount was restored (Fig. 4A). No significant differences were found in cell proliferation between 2D and Re-2D cultures.

To further study the cell cycle activity, expression of *Ccna2* (*Cyclin A*) and *Ccne1* (*Cyclin E*), essential genes for cell cycle progression, were compared between 2D, 3D, and Re-2D cultures (Fig. 4B). It was revealed that *Cyclin A* expression was significantly decreased in 3D cultures compared with 2D cultures. When the stellate-shaped cells in 3D cultures were recovered and plated back in 2D cultures (Re-2D), the *Cyclin A* expression increased significantly compared to 3D cultures. Similarly, the expression of *Cyclin E* in 3D cultures was lower than in 2D and Re-2D cultures, although no significant differences were detected. In MC3T3-E1 cell cultures, the change in DNA amounts was almost the same as that observed in primary osteoblast cultures (Fig. 4C). Consistent with this finding, the expression of *Cyclin A* and *E* was lower in 3D cultures compared to 2D and Re-2D cultures (Fig. 4D). These findings suggest that when osteoblasts are cultured in 3D conditions, the cells become dormant as their morphology shifts from fibroblast-like to stellate. Dormant cells in 3D cultures can then become proliferatively active when cultured back in 2D conditions (Re-2D).

### Calcium deposition is suppressed in 3D cultures but substantial in Re-2D cultures

**Figure 5:**
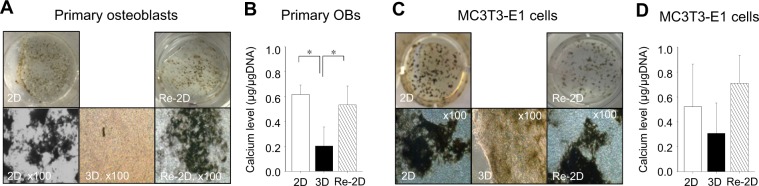
Calcium deposition. Representative photomicrographs of Von Kossa-stained primary osteoblast cultures (**A**) and MC3T3-E1 cell cultures (**C**). Cells were cultured for 21 days in osteogenic medium under 2-dimensional (2D) or 3-dimensional (3D) conditions. A separate group of cells in 3D cultures for 10 days was recovered and plated back in 2D cultures for another 21 days (Re-2D) in osteogenic medium. Calcium deposits in the extracellular matrix were identified with Von Kossa staining. Calcium levels normalised by total DNA were statistically analysed using a one-way ANOVA (**B**,**D**). *p < 0.05.

To examine the influence of culture conditions on calcium deposition, cells were cultured in osteogenic medium, and calcium deposits were quantified. The deposits visualised by Von Kossa and Alizarin red staining are shown in Fig. 5 and Supplementary Fig. S2, respectively. The findings obtained with Alizarin red staining were nearly the same as those with Von Kossa staining. Since collagen gel matrices in 3D cultures resulted in a high staining background, thin slices of the gels (approximately 1 mm in thickness) were made and photomicrographed.

When primary osteoblasts were cultured in 2D osteogenic conditions, calcium deposition was observed as expected (Fig. 5A). Moreover, the calcium deposition in 3D osteogenic cultures was significantly lower than that in 2D osteogenic cultures (Fig. 5B).

To determine whether or not cells that had taken on a stellate shape in 3D cultures could deposit calcium once plated back in 2D osteogenic conditions (Re-2D), we first induced stellate-shaped cells in 3D cultures and then plated them back in Re-2D under osteogenic conditions (Fig. 1C). After 21 days of Re-2D osteogenic culture, significantly higher calcium deposits were detected in Re-2D cultures than those in 3D osteogenic cultures. No marked differences were found in calcium deposits between 2D and Re-2D osteogenic cultures.

Similarly, in MC3T3-E1 cells, calcium deposits in 3D osteogenic cultures were not as high as those in 2D osteogenic cultures (Fig. 5D). When cells were recovered from 3D cultures and plated back in 2D osteogenic conditions (Re-2D osteogenic), a considerable amount of calcium deposition was found. Therefore, cells in 3D cultures appeared to have limited mineralisation activity. However, once they were cultured back in 2D osteogenic conditions, the cells actively mineralised ECM.

## Discussion

It is well accepted that osteocytes and bone-lining cells are terminally differentiated cells derived from mature osteoblasts. The literature indicates that bone-lining cells are able to dedifferentiate back into mature osteoblasts without genetic manipulation and contribute to the repair of bone^16–18^. However, whether or not osteocytes are capable of dedifferentiating back into mature osteoblasts and regaining their proliferation and mineral depositing properties has been unclear. The results of this study show that osteocytes are indeed able to dedifferentiate back into mature osteoblasts without genetic manipulation. Mature osteoblasts in 2D cultures differentiated into osteocytes when grown in 3D cultures, and those osteocytes dedifferentiated back into osteoblasts when grown in 2D cultures.

It has been demonstrated that osteoblasts differentiate into osteocytes in the 3D culture systems *in vitro*^19–22^. Those previous studies reported that the cell morphology shifted from a typical fibroblast-like form into a stellate shape, and the expression of osteocyte marker genes, such as *Sost* and *Dmp1*, was elevated. Our results showing a considerable change in the cell morphology and a gene expression profile in 3D cultures are consistent with the findings of these previous reports. We further confirmed that the stellate-shaped cells in 3D cultures were in cell cycle arrest, indicating these cells entered into a quiescent state.

However, very little is known about the dedifferentiation of osteocytes back into osteoblasts. Using osteocyte-specific transgenic mice, in which osteocytes express the tdTomato reporter (Ai9), Torreggiani *et al*. reported that osteocytes (Ai9^+^) in the lacunae exited the bone and dedifferentiated into the osteoblastic phenotype *in vitro* and *in vivo*^23^. The study showed that dedifferentiated osteoblasts deposited mineral in osteogenic 2D cultures. However, the expression of *Dmp1* in the dedifferentiated cells increased during osteogenic differentiation in that study. Considering that mature osteoblasts hardly express *Dmp1*^2^, the increased expression of *Dmp1* in 2D cultures may imply that these cells were not fully dedifferentiated. In our study, osteoblasts differentiated into osteocytes in 3D cultures. The cells in 3D cultures expressed high levels of *Dmp1*, *Fgf23*, and *Sost* and low levels of *Alpl*. They were also in cell cycle arrest and had low mineralisation propensity. These osteocytes dedifferentiated back into osteoblasts in Re-2D cultures, expressing low levels of *Dmp1*, *Fgf23*, and *Sost* and high levels of *Alpl*. They also exhibited a high propensity to mineralise.

It was once perceived that cell differentiation is unidirectional in humans. However, accumulating evidence indicates that differentiated cells have the potential to dedifferentiate or transdifferentiate into other specialized cells or immature progenitors without genetic manipulation^24–27^. Remarkable plasticity exists in cells in the mesenchymal lineage^28,29^. Mesenchymal stem cells (MSCs) give rise to adipocytes, osteoblasts, chondrocytes, and other cells^30^. In bone biology research, cells from cultured bone fragments are often used as osteoblasts due to their high mineralisation propensity. Considering that osteocytes constitute over 95% of cells in bone, it is reasonable to think that a sizeable portion of cells from bone fragment outgrowth is derived from osteocytes. Cells from bone fragment outgrowth cultures have been shown to have the capacity to differentiate into osteoblasts, adipocytes, and chondrocytes^28,31^. Zhu *et al*. reported that cells that exited bone fragments in cultures were predominantly MSCs^32^. These bone fragment-derived MSCs have the multi-lineage differentiation capacity to undergo adipogenesis, chondrogenesis, and osteogenesis. Ullah *et al*. investigated whether or not dedifferentiated cells in the mesenchymal lineage carry this multi-lineage differentiation capacity^33^. Their study showed that cells dedifferentiated from adipocytes were able to undergo osteogenic, adipogenic, and chondrogenic differentiation. Thus, “terminally” differentiated cells may be a misnomer since such cells in the mesenchymal lineage maintain cellular plasticity.

When adherent cells are cultured in a 3D condition, the cell morphology is generally different from that in a 2D condition. Cells in 3D cultures typically develop long processes and interconnect with neighbouring cells to form a network. It has been demonstrated that capillary endothelial cells develop a network of anastomosing vessel-like structures when grown in 3D cultures, while they grow as typical monolayers without forming tubular structures in 2D cultures^34^. Likewise, fibroblasts in 3D cultures extend their processes to form a dendritic network not seen in 2D cultures^35^. Furthermore, changes in cell morphology typically accompany changes in cell phenotypes^36^. Thus, the modification of the cellular microenvironment has considerable influence on cell phenotypes. Our findings, which showed the dedifferentiation of osteocytes back into osteoblasts, were likely induced by the modification of the cellular microenvironment.

In summary, when the cellular microenvironment was switched between 2D and 3D, not only morphological changes but also changes in the gene expression profiles were noted, shifting between osteoblasts and osteocytes. The cell status shifted between quiescent and active as well, and changes in the mineral deposition capability of cells between 2D and 3D conditions were also noted. Taken together, the results of this study indicate that osteocytes have the capacity to dedifferentiate back into mature matrix producing osteoblasts.

## Material and Methods

### Animals and primary osteoblasts

Three breeding pairs of mice (C57BL/6) were purchased from a commercial vendor (Kyudo, Fukuoka, Japan) and maintained in-house. The animal experimental protocol (#17018) was approved, and all animals were treated according to the guidelines of the Committee on Use and Care of Animals of Fukuoka Dental College.

Primary osteoblasts were isolated from the calvariae of neonates following a protocol previously used^37^. In brief, the calvariae of neonatal mice were dissected and subjected to 5 sequential 30-min digestions in collagenase A with 0.25% trypsin at 37 °C. Cell fractions from the third to fifth digestion were collected and cultured in αMEM supplemented with 10% fetal bovine serum, 1% GlutaMAX-I, and 1% penicillin-streptomycin.

### 2D and 3D cell cultures

Murine osteoblast cell line MC3T3-E1 clone-4 cells and primary osteoblasts were cultured in αMEM for 10 days. Cells were harvested, washed with phosphate-buffered saline (PBS), and plated at 1.0 × 10^5^ cells/cm^2^ in αMEM for another 10 days with fresh medium replacement every 2 days (2D culture). Alternatively, cells were maintained in a 3D culture system in 12-well plates. The system comprised three layers: the bottom collagen gel layer, the middle collagen gel layer, and the top culture medium layer (Fig. 1A). Collagen gels were made of 0.12% Cellmatrix Type I-A (Nitta Gelatin, Osaka, Japan) dissolved in αMEM. The bottom gel layer (0.5 mL) was cell-free. The middle gel layer (1.0 mL) contained cells (1.0 × 10^5^ cells/mL). The top layer was αMEM (1.0 mL). Cells were cultured in 3D conditions for 10 days with fresh medium replacement every 2 days (3D culture). To recover cells from 3D cultures, gels were minced and digested with collagenase type I. Recovered cells were neutralised, washed with PBS, and plated at 1.0 × 10^5^ cells/cm^2^ in αMEM for another 10 days with fresh medium replacement every 2 days (Re-2D culture). Cells were maintained in 5% CO_2_ at 37 °C.

### Morphological assessment

Cells in 2D, 3D, and Re-2D cultures (Fig. 1B) were stained to visualize nucleus and actin. Cells were fixed with 4% paraformaldehyde, washed, and incubated with 70% ethanol to increase membrane permeability. Phalloidin (1:1000) and DAPI (1:400) were applied together for 40 min in the dark at 4 °C. Cells were photomicrographed using a fluorescent microscope equipped with a camera (Ti2-U with DS-Fi1c, Nikon, Tokyo, Japan). More than 20 cells were randomly selected from the images of each culture, and the size of the cell bodies and the length of the dendrites were quantified using the ImageJ software program.

### Quantitative polymerase chain reaction (qPCR)

Total RNA was extracted from cells in 2D and Re-2D cultures using the TRIzol reagent according to the manufacturer’s protocol. For cells in 3D cultures, gels were first crushed with TRIzol in liquid nitrogen, and then total RNA was extracted with additional TRIzol. cDNA was synthesised from 2 µg of total RNA using the PrimeScript RT reagent Kit (Takara, Osaka, Japan). cDNA was used as the template for qPCR to quantify the expression of *Alpl*, *Sp7* (*Osterix*), *Sost*, *Fgf23*, *Dmp1*, *Tnfsf11* (*Rankl*), *Tnfrsf11b* (*Opg*), *Ccna2* (*Cyclin A*), and *Ccne1* (*Cyclin E*). Assays were carried out using the Thunderbird qPCR Mix (Toyobo, Shiga, Japan) and AriaMx Real-Time PCR System (Agilent, Santa Clara, CA, USA). All samples were run in triplicate to control for PCR variation. The expression was calculated using the standard curve method. An internal control gene, *Gapdh*, was used to normalise the expression of target genes. The amount of the *Gapdh* expression per 2 µg of total RNA was measured to compare its expression between 2D, 3D, and Re-2D cultures. Each experiment was repeated at least three times using independent RNA samples. Primer sequences are listed in Supplementary Table S1.

### Quantification of DNA

DNA amounts were quantified at days 1, 3, 5, 7, and 10 in 2D, 3D, and Re-2D cultures in order to assess cell proliferation. DNA was extracted using the TRIzol reagent according to the manufacturer’s protocol. The DNA concentration was determined with NanoDrop Lite (Thermo Scientific, Wilmington, DE, USA).

### Assessment of calcium deposits

Freshly isolated primary osteoblasts and MC3T3-E1 cells were maintained in αMEM for 10 days. Cells were harvested, washed with PBS, and seeded at 1.0 × 10^5^ cells/cm^2^ for 2D or at 1.0 × 10^5^ cells/mL for 3D cultures in αMEM. At day 3, the medium was replaced with osteogenic medium (αMEM, 50 μg/ml ascorbic acid, 10 mM β-glycerophosphate) and cultured for another 18 days to stimulate calcium deposition (Fig. 1C). For Re-2D cultures, cells were cultured in the 3D condition in αMEM for 10 days. Cells were then recovered from the 3D cultures and re-plated at 1.0 × 10^5^ cells/cm^2^ in αMEM for Re-2D cultures. At day 3, the medium was replaced with osteogenic medium and cultured for another 18 days.

Von Kossa or Alizarin red staining was performed to visualise calcium deposits. For Von Kossa, cells were fixed in ethanol followed by incubation with a 5% silver nitrate solution in the dark, exposed to bright light and stained calcium deposits were quantified. Cells were washed, scraped, or minced (3D) with 10 mM Tris-HCL (pH 7.4) and then mixed with an equal volume of 30% trichloroacetic acid overnight. Insoluble materials were removed by low-speed centrifugation. Supernatants were assayed for calcium with a commercially available kit (Pointe Scientific, Canton, MI, USA). DNA in pellets was extracted with the TRIzol method and quantified using NanoDrop. Calcium data were normalized to cellular DNA.

### Statistical analyses

All statistical analyses were performed using the SYSTAT 12 software program (Systat, San Jose, CA, USA). Data were expressed as the mean ± standard deviation. A one-way analysis of variance (ANOVA) was performed to statistically compare differences between the 2D, 3D, and Re-2D cultures for the morphological assessment, gene expression, and calcium deposition. A two-way ANOVA was performed to compare differences in the amounts of DNA over time between 2D, 3D, and Re-2D cultures. Tukey’s test was used for post-hoc comparisons. A *p*-value of less than 0.05 was considered significant.

## Supplementary information

Supplemental_Information

## Data Availability

All data generated or analysed during this study are included in this published article and its Supplementary Information files.
