# Author Correction: Alternating Differentiation and Dedifferentiation between Mature Osteoblasts and Osteocytes

**DOI:** 10.1038/s41598-020-78856-4

**Published:** 2020-12-07

**Authors:** Naruhiko Sawa, Hiroki Fujimoto, Yoshihiko Sawa, Junro Yamashita

**Affiliations:** 1 Department of Oral Rehabilitation, Fukuoka Dental College, Fukuoka, Japan; 2 Department of Oral Function and Anatomy, Okayama University Graduate School of Medicine, Dentistry and Pharmaceutical Sciences, Okayama, Japan; 3 Center for Regenerative Medicine, Fukuoka Dental College, Fukuoka, Japan

Correction to: *Scientific Reports* 10.1038/s41598-019-50236-7, published online 25 September 2019

This Article contains errors.

As a result of an error during figure assembly, in Figure 5C the culture image for sample Re-2D is incorrect. The correct Figure 5 is included below as Figure 1.

**Figure 1:**
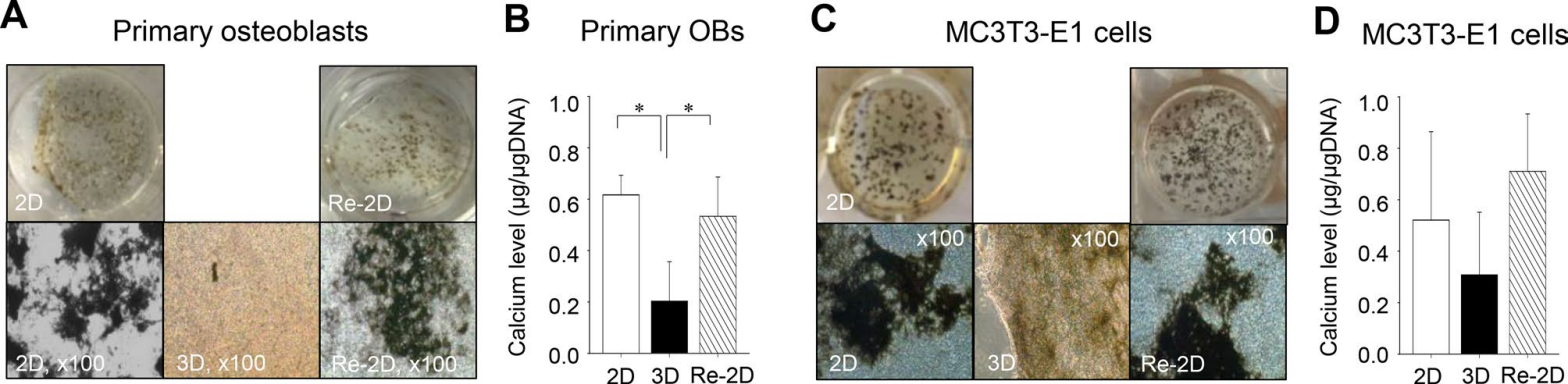
Calcium deposition. Representative photomicrographs of Von Kossa-stained primary osteoblast cultures (**A**) and MC3T3-E1 cell cultures (**C**). Cells were cultured for 21 days in osteogenic medium under 2-dimensional (2D) or 3-dimensional (3D) conditions. A separate group of cells in 3D cultures for 10 days was recovered and plated back in 2D cultures for another 21 days (Re-2D) in osteogenic medium. Calcium deposits in the extracellular matrix were identified with Von Kossa staining. Calcium levels normalised by total DNA were statistically analysed using a one-way ANOVA (**B**,**D**). *p < 0.05.

This change does not affect the conclusions of the Article.

