# Supplementary material for: Alternating Differentiation and Dedifferentiation between Mature Osteoblasts and Osteocytes
Source: Sci Rep. 2019 Sep 25;9:13842. doi: 10.1038/s41598-019-50236-7 (PMC6761144; doi:10.1038/s41598-019-50236-7)
Supplement: Supplementary file 1 — Supplemental_Information [file 41598_2019_50236_MOESM1_ESM.pdf]

Supplemental information

Alternating Differentiation and Dedifferentiation between Mature Osteoblasts and Osteocytes

Naruhiko Sawa, Hiroki Fujimoto, Yoshihiko Sawa, *Junro Yamashita

Suppl. Table S1. qPCR Primers

Gene	Forward primer sequence (5'-3')	Reverse primer sequence (5'-3')
Alpl	AGGCAGGATTGACCACGG	TGTAGTTCTGCTCATGGA
Sp7	TGCTTGAGGAGGAAGTTCAC	AGGTCACTGCCCACAGAGTA
Sost	CTTCAGGAATGATGCCACAGAGGT	ATCTTTGGCGTCATAGGGATGGTG
Fgf23	ACTTGTCGCAGAAGCATC	GTGGGCGAACAGTGTAGAA
Dmp1	GGCTGTCCTGTGCTCTCCCAG	GGTCACTATTTGCCTGTGCCTC
Tnfsf11	CAAGCTCCGAGCTGGTGAAG	CCTGAACTTTGAAAGCCCCA
Tnfrsf11b	AAGAGCAAACCTTCCAGCTGC	CACGCTGCTTTCACAGAGGTC
Gapdh	ACCCAGAAGACTGTGGATGG	CAGATTGGGGGTAGGAACAC
Ccna2	CCTGCCTTCACTCATTGCTG	GTGGCGCTTTGAGGTAGGT
Ccne1	GTGTCAAATGGATGGTTC	GGAGAAATCCTATTCTGTTC

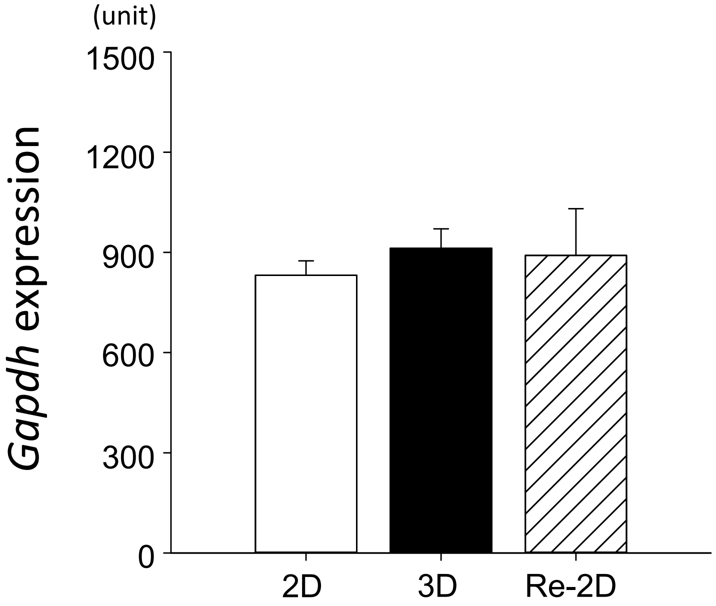

Suppl. Fig. S1. The amount of the *Gapdh* expression per 2 μg of total RNA. No significant differences were noted between 2D, 3D, and Re-2D conditions.

Alizarin red staining

Cells were cultured in osteogenic medium (αMEM, 50 µg/ml ascorbic acid, 10 mM β-glycerophosphate) to stimulate calcium deposition (Figure 1C). Alizarin red staining was performed to visualize calcium deposits. At the end of cell culture in osteogenic medium, cells were fixed in 70% ethanol followed by incubation with 40 mM Alizarin red, pH 4.2, for 10 minutes. Cell cultures were washed to remove excess alizarin red staining.

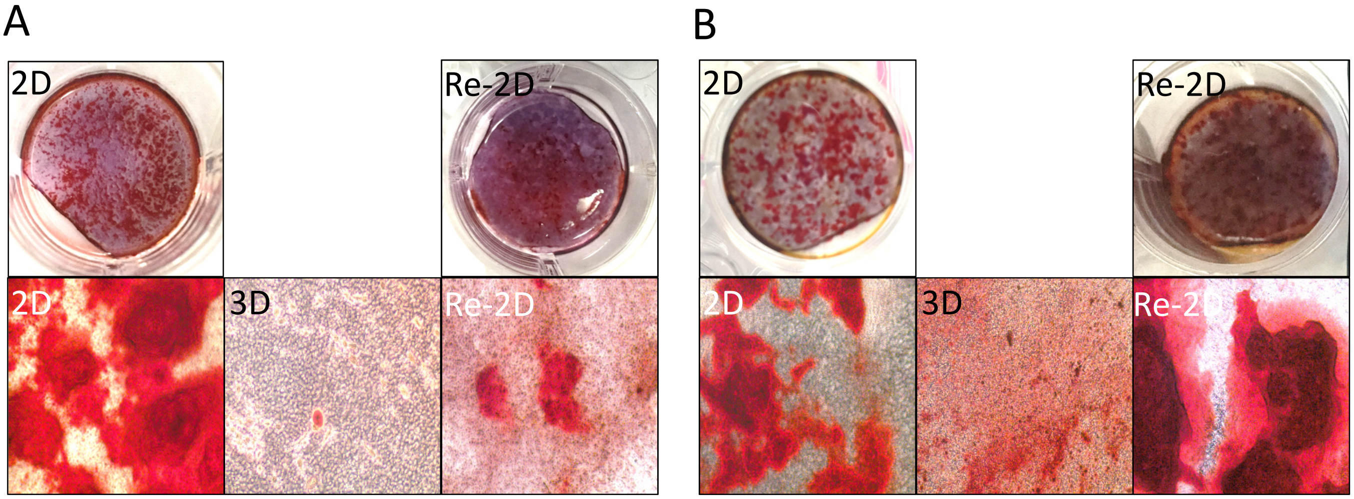

Suppl. Fig. S2. Representative photomicrographs of Alizarin red stained calcium deposits. Primary osteoblast cultures (A) and MC3T3-E1 cell cultures (B) are shown.
